# Locating influential nodes via dynamics-sensitive centrality

**DOI:** 10.1038/srep21380

**Published:** 2016-02-24

**Authors:** Jian-Guo Liu, Jian-Hong Lin, Qiang Guo, Tao Zhou

**Affiliations:** 1 Data Science and Cloud Service Centre, Shanghai University of Finance and Economics, Shanghai 200433, PR China; 2 Research Center of Complex Systems Science, University of Shanghai for Science and Technology, Shanghai 200093, PR China; 3 CompleX Lab, Web Sciences Center, University of Electronic Science and Technology of China, Chengdu 611731, PR China

## Abstract

With great theoretical and practical significance, locating influential nodes of complex networks is a promising issue. In this paper, we present a dynamics-sensitive (DS) centrality by integrating topological features and dynamical properties. The DS centrality can be directly applied in locating influential spreaders. According to the empirical results on four real networks for both susceptible-infected-recovered (SIR) and susceptible-infected (SI) spreading models, the DS centrality is more accurate than degree, *k*-shell index and eigenvector centrality.

Spreading dynamics represents many important processes in nature and society^1^,^2^, such as the propagation of computer viruses^3^ and traffic congestion^4^, reaction diffusion^5^, spreading of infectious diseases^6^ and cascading failures^7^. The estimation of nodes’ spreading influences can help in hindering epidemics or accelerating innovation^8^, and similar methods can be further applied in identifying influential spreaders in social networks^9^, quantifying the influence of scientists and their publications^10^, evaluating the impacts of injection points in the diffusion of microfinance^11^, finding drug targets in directed pathway networks^12^, predicting essential proteins in protein interaction networks^13^, and so on.

The significance of this issue triggers a variety of novel approaches in identifying influential spreaders in networks, which can be roughly categorized into three classes. Firstly, some scientists argued that the location of a node is more important than its immediate neighbors, and thus proposed *k*-shell index^14^,^15^ and its variants^16^,^17^,^18^,^19^ as indicators of spreading influences. Secondly, some scientists quantified a node’s influence only accounting for its local surroundings^20^,^21^,^22^. Thirdly, some scientists evaluated nodes’ influences according to the steady states of some introduced dynamical processes, such as random walk^23^,^24^ and iterative refinement^25^.

The above-mentioned approaches only take into account the topological features, while recent experiments indicate that the performance of structural indices is very sensitive to the specific dynamics on networks^26^,^27^,^28^. For example, when the spreading rate is very small, the degree usually performs better than the eigenvector centrality^29^ and *k*-shell index^14^, while when the infectivity is very high, the eigenvector centrality is the best one among the three (see Figs 1 and 2, with details shown later). To the best of our knowledge, there are few works taking into account the properties of the underlying spreading dynamics^30^,^31^,^32^. Encoding the interplay between topology and dynamics, Klemm *et al.*^30^ suggested that the eigenvector centrality can be used in estimating nodes’ dynamical influences in the susceptible-infected-recovered (SIR) spreading model (also called susceptible-infected-removed model)^33^. Li *et al.*^31^ provided complementary explanation of the suitability of eigenvector centrality based on perturbation around the equilibrium of the epidemic dynamics and discussed the limitations of eigenvector centrality for homogeneous community networks. Both the above two works did not pay enough attention to the specific parameters in the spreading models, and thus their suggested index only works well in a limited range of the parameter space. Bauer and Lizier^32^ proposed an approach based on counting the number of possible infection walks with different lengths to approximate the number of infected nodes generated by a given initially infected node. Their method is an effective one to predict the outbreak size over a wide range of spreading rate but less efficient due to the considerable computational cost. In addition, for the fundamental complexity in counting the number of paths connecting two nodes, their method could not be formulated in a compact analytical form.

In this paper, we describe the infectious probabilities of nodes by a matrix differential function that accounts both topological features and dynamical properties. Accordingly, we propose a dynamics-sensitive (DS) centrality to predict the outbreak size at given time step, which can be directly applied in quantifying the spreading influences of nodes. According to the empirical results on four real networks, for both the SIR model^33^ and the susceptible-infected (SI) model^34^,^35^, the DS centrality can locate influential nodes accurately and performs particularly well in the early stage of spreading. The method proposed in this paper can be extended to other Markov processes on networks.

## Dynamics-Sensitive Centrality

A simple undirected connected network *G* = (*V*, *E*) with 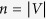 nodes and 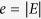 links could be described by an adjacency matrix 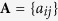 where *a*_*ij*_ = 1 if node *i* is connected with node *j*, and *a*_*ij*_ = 0 otherwise. **A** is binary and symmetric with zeros along the main diagonal, and thus its eigenvalues are real and can be arrayed in a descending order as 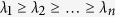. Since **A** is a symmetric and real-valued matrix, it can be factorized as 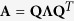, where 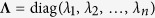, 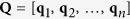 and **q**_*i*_ is the eigenvector of the eigenvalue *λ*_*i*_.

We consider a discrete-time spreading model where an infected node would infect its neighbors with spreading rate *β* and recover with recovering rate *μ* (see **Materials and Methods** for details). Denote **x**(*t*) (*t* > 0) as an approximation of the cumulative probabilities that nodes are excited between time step 1 and *t*, and then **x**(*t*) − **x**(*t* − 1) (*t* > 1) is approximated as the probabilities of nodes to be infected at time step *t*. If *i* is the only initially infected node, then *x*_*i*_(0) = 1 and *x*_*j*≠*i*_(0) = 0. In the first time step, **x**(1) = *β***Ax**(0), and for *t* > 1, we have (see the derivation in **Materials and Methods**)


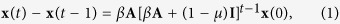


where **I** is the identity matrix. Denoting 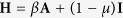, then 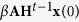 represents the probabilities of nodes to be infected at time step *t*, and thus the cumulative probabilities of nodes to have been infected between time step 1 and *t* can be approximated as


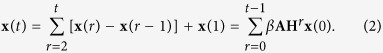


We define *S*_*i*_(*t*) to be the spreading influence of node *i* at time step *t*, which can be quantified by the sum of infected probabilities of all nodes, given *i* the initially infected seed. According to Eq. (2), the infected probabilities can be written as


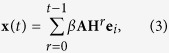


where **e**_*i*_ = (0, …, 0, 1, 0, …, 0)^*T*^ is an *n* × 1 vector with only the *i*th element being 1. As all elements other than the *i*th one of **e**_*i*_ are zero, **x**(*t*) is indeed the sum of all the *i*th columns of 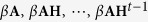. Given **x**(0) = **e**_*i*_, *S*_*i*_(*t*) is defined as the sum of all elements of **x**(*t*), which is equal to the sum of all elements in the *i*th columns of 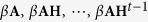, as


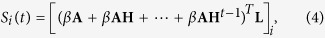


where **L** = (1, 1, …, 1)^*T*^ is an *n* × 1 vector whose components are all 1. Obviously, **A**^*T*^ = **A**, **H**^*T*^ = **H** and **AH** = **HA**, so the spreading influence of all nodes can be described by the vector


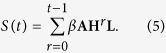


Notice that, 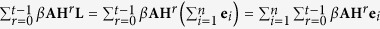, and 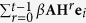 is the infected probabilities of all nodes given node *i* the only initially infected seed according to Eq. (2), so **S**(*t*) can also be roughly explained as the sum of infected probabilities over the *n* cases with every node being the infected seed once. This relationship shows an underlying symmetry, that is, in an undirected network, the node having higher influence is also the one apt to be infected. The readers are warned that such conclusion is not mathematically rigorous since we have ignored the complicated entanglement by allowing the elements of **x**(*t*) being larger than 1.

The eigenvectors of **H** are the same to the ones of **A** and *βλ*_*i*_ + 1 − *μ* is the *i*th eigenvalue of **H**, corresponding to **q**_*i*_. When *βλ*_1_ + 1 − *μ* < 1, i.e. *β*/*μ* < 1/*λ*_1_ (for the case *μ* ≠ 0), **H**^*t*^**L** could converge to null vector when *t*→∞ and **S**(*t*) could be written by the following way


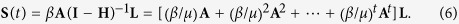


For simplicity, in the SIR model, we set *μ* = 1, and then


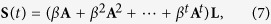


where (**A**^*t*^**L**)_*i*_ counts the total number of walks of length *t* from node *i* to all nodes in the network, weighted by *β*^*t*^ that decays as the increase of the length *t*. As **S**(*t*) quantifies nodes’ spreading influences, we call it dynamics-sensitive (DS) centrality, where the term *dynamics-sensitive* emphasizes the fact that **S**(*t*) is determined not only by the network structure (i.e., **A**), but also the dynamical parameters (i.e., *β* and *t*). In particular, when *t* = 1, the initially infected node only has the chance to infect its neighbors and *S*_*i*_(1) = (*β***AL**)_*i*_ with (**AL**)_*i*_ being exactly the degree of node *i*. When *μ* = 0 (corresponding to the SI model) or *β* ≥ 1/*λ*_1_, **S**(*t*) would be infinite when *t*→∞, which could not reflect the spreading influences. In fact, there are two underlying assumptions. Firstly, the DS centrality calculates the probabilities of the nodes to be infected at time step *t* by linear coupling instead of nonlinear coupling. For example, if a susceptible node has *m* infected neighbors, the probability of the node to be infected is approximated as *mβ* by the DS centrality, instead of 1 − (1 − *β*)^*m*^, which is the exact probability of the node to be infected. Secondly, the DS centrality calculates the probabilities of nodes to be infected under the assumption that the nodes are susceptible at time steps *t*. Notice that, our main goal is to find out the ranking of spreading influences of nodes, namely to identify influential nodes. As every node’s infected probability is overestimated, the ranking may be less affected. Thus far, we are still not aware of the impacts on the ranking, while fortunately, as later indicated by the extensive numerical results, the DS centrality performs much better than other well-known indices for a very broad ranges of *β* and *t* that cover most practical scenarios.

## Results

We test the performance of DS centrality in evaluating the nodes’ spreading influences according to the SIR model and SI model, with varying spreading rate *β*. Four real networks, including a scientific collaboration network, an email communication network, the Internet at the router level and a protein-protein interaction network, are used for the empirical analysis (see data description in **Materials and Methods**), and three well-known indices, including degree, *k*-shell index and eigenvector centrality, are used as benchmark methods for comparison (see **Materials and Methods** for the definitions of those indices). Given the time step *t*, the spreading influence of an arbitrary node *i* is quantified by the number of infected nodes (for SI model) or the number of infected and recovered nodes (for SIR model) at *t*, where the spreading process starts with only node *i* being initially infected. Here we use Kendall’s Tau *τ*^36^ to measure the correlation between nodes’ spreading influences and the considered centrality measure, where *τ* is in the range [−1, 1] and the larger *τ* corresponds to the better performance (see **Materials and Methods** for the definition of *τ*).

**Figure 1:**
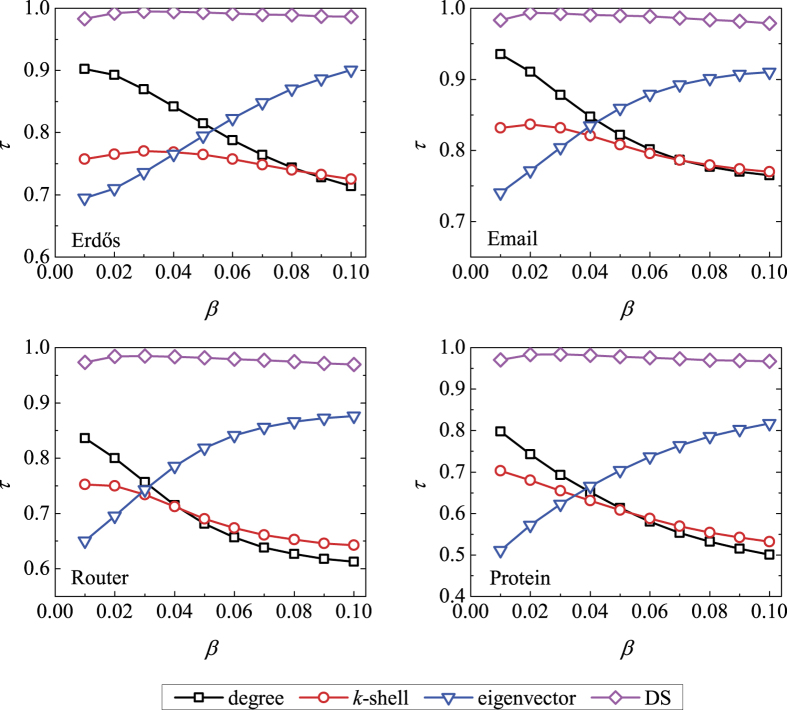
The accuracy of four centrality measures in evaluating nodes’ spreading influences according to the SIR model (*μ* = 1) in the four real networks, quantified by the Kendall’s Tau. The spreading rate *β* varies from 0.01 to 0.10, and the time step is set as *t* = 5. Each data point is obtained by averaging over 10^4^ independent runs.

**Figure 2:**
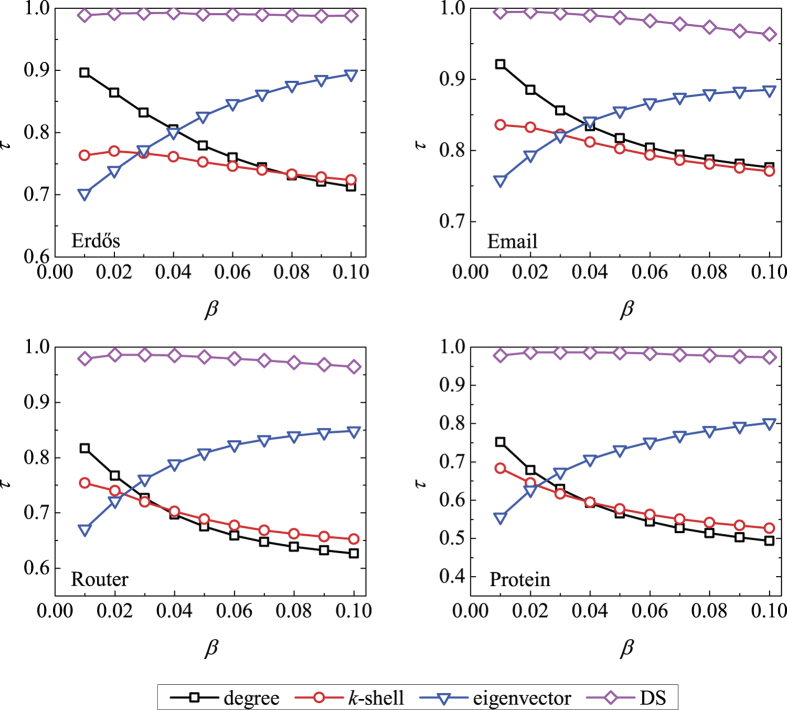
The accuracy of four centrality measures in evaluating nodes’ spreading influences according to the SI model (*μ* = 0) in the four real networks, quantified by the Kendall’s Tau. The spreading rate *β* varies from 0.01 to 0.10, and the time step is set as *t* = 5. Each data point is obtained by averaging over 10^4^ independent runs.

As shown in Fig. 1, the Kendall’s Tau *τ* for the DS centrality is between 0.968 and 0.995 for 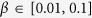, indicating that the ranking lists generated by the DS centrality and the real SIR spreading process are highly identical to each other. In comparison, the DS centrality performs better than degree, *k*-shell index and eigenvector centrality since the DS centrality takes into account one more parameter *β*. As shown in Fig. 2, similar results are also observed for the SI model where the DS centrality performs better than others. The results for larger *β* and *t* are respectively shown in Figs S1 and S2 of Supplementary Information, where the DS centrality still could locate influential nodes accurately. Meanwhile, Fig. S3 shows that the DS centrality also performs much better than other compared indices in evaluating the nodes’ spreading influences for *μ* = 0.3 and 0.7 in the SIR model.

Since **A** is a symmetric, real-valued matrix, the DS centrality **S**(*t*) can be written in the following way by decomposing **A**


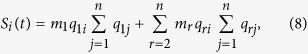


where 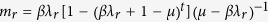 for 1 ≤ *r* ≤ *n*. Rewriting Eq. (8) into


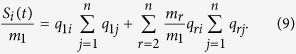


With the increase of *t* and *β*, 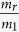 will converge to 0, and thus the ranking lists generated by **S**(*t*) will be identical to **q**_1_, which is exactly the same to the eigenvector centrality. This relationship is in accordance with the results presented in Figs S1 and S2, where the difference between the eigenvector centrality and DS centrality gets smaller as the increase of *β* and *t*.

## Conclusion and Discussions

Estimating the spreading influences and then identifying influential nodes are fundamental task before any regulation on the spreading process. For such task, most known works only took into account the topological information^8^. Recently, Aral and Walker^37^ showed that the attributes of nodes are highly correlated with nodes’ influences and tendencies to be influenced. In this paper, in addition to the topological information, we get down to the underlying spreading dynamics and propose a dynamics-sensitive (DS) centrality, which is a kind of weighted sum of walks ending at the target node, where both the spreading rate and spreading time are accounted in the weighting function. The DS centrality can be directly applied in quantifying the spreading influences of nodes. According to the empirical analyses of the SIR model and SI model at given time step on four real networks, the DS centrality can locate influential nodes much more accurately than degree, *k*-shell index and eigenvector centrality. In fact, it is unfair to directly compare the DS centrality with the above structure-based indices since the DS centrality takes into account one more parameter *β*, while the present result is still significant as it gives an elegant way to account the specific dynamics and shows the remarkable improvement by introducing the parameter *β*, which may be expected but not yet well demonstrated before this paper.

The DS centrality performs particularly well in the early stage of spreading, which provides a powerful tool in early detection of potential super-spreaders for epidemic control. Notice that, we did not consider the asymptotical case with *t*→∞, since after sufficiently long spreading time, all nodes in the network would be infected under large spreading rate, especially in the SI model. In such case, different nodes spreading influences are not distinguishable.

The DS centrality tells us an often ignored fact that the most influential nodes are dependent not only on the network topology but also on the spreading dynamics. Given different models and parameters, the relative influences of nodes are also different. Roughly speaking, if the spreading rate is small, we can focus on the close neighborhood of a node since it is not easy to form a long spreading pathway (i.e., *β*^*t*^ decays very fast as the increase of *t* when *β* is small) while if the spreading rate is high, the global topology should be considered. A clear limitation of this work is that before calculating the DS centrality, we have to know the spreading rate that is usually a hidden parameter. This parameter can be effectively estimated according to the early spreading process^38^ and then we can calculate the DS centralities by varying the spreading rates over the estimated range and see which nodes are the most influential ones in average. In addition to the identification of the most influential node, a related but more challenging problem is to find the most influential sets of nodes^39^. The DS centrality could not be directly extended to solve this problem, however, similar to the present idea, the dynamical information is very necessary in dealing with this problem^40^.

Some other centralities related to specific dynamical processes have also been proposed recently, including routing centrality^41^, epidemic centrality^42^, diffusion centrality^43^, percolation centrality^44^ and game centrality^45^. Comparing with these centralities, similar to the works by Klemm *et al.*^30^,^46^,^47^, this paper provides a more general framework that could deal with other Markov processes and thus can be extended and applied in many other important dynamics, such as the Ising model^48^, Boolean dynamics^49^, voter model^50^, synchronization^51^, and so on. For example, Zhou *et al.*^35^ considered a variant SIR spreading model where an infected node can only contact one neighbor in one time step. In this case, the adjacency matrix **A** in the DS centrality could be replaced by **D**^−1^ **A**, where **D** is an *n* × *n* matrix where *i*th main diagonal element is the degree of node *i* and other elements are 0. Then the DS centrality can be extended to this spreading model. Furthermore, the DS centrality can also be directly extended to asymmetrical networks and weighted networks. We hope this work could highlight the significant role of underlying dynamics in quantifying the individual nodes’ importance, and then the difference between lists of critical nodes for different dynamical processes could be considered as their distinguished properties.

## Materials and Methods

### Derivation of Eq. (1)

The probabilities of nodes to be infected at time step *t* = 2 can be approximated as


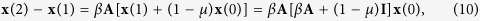


where **x**(1) + (1 − *μ*)**x**(0) is the probabilities that nodes still have the ability to infect the susceptible nodes at time step 2. We assume that when *t* ≤ *p*, 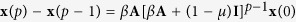, then for *t* = *p* + 1, we have


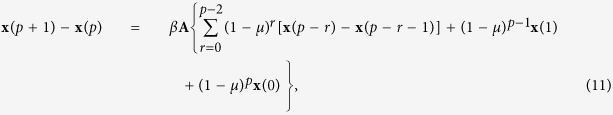


where 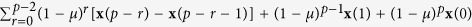 is the probabilities that nodes still have the ability to infect the susceptible nodes at time step *p* + 1. Therefore, we can get that


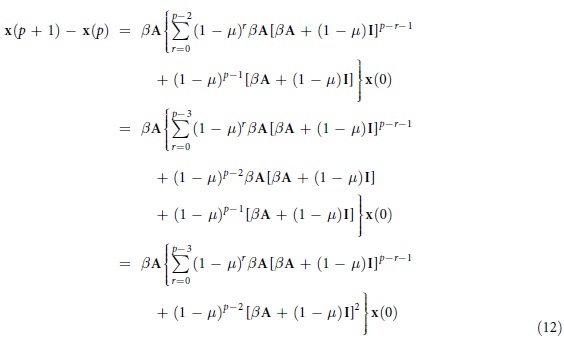



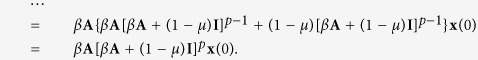


Therefore, according to the mathematical induction, Eq. (1) is established.

### Spreading Model

Here we apply the susceptible-infected-recovered (SIR) model (also called the susceptible-infected-removed model)^33^. In the SIR model, there are three kinds of individuals: (i) susceptible individuals that could be infected, (ii) infected individuals having been infected and being able to infect susceptible individuals, and (iii) recovered individuals that have been recovered and will never be infected again. In this paper, the spreading process starts with only one seed node being infected initially, and all other nodes are initially susceptible. At each time step, each infected node makes contact with its neighbors and each susceptible neighbor is infected with a probability *β*. Then each infected node enters the recovered state with a probability *μ*. In the main text we set *μ* = 1, and for other values of *μ*, the results are very similar (see SI). In the standard SI model, nodes can only be susceptible or infected, corresponding to the case with *μ* = 0. In this paper, the model, analysis and simulations are all based on the discrete-time dynamics.

### Benchmark Methods

The degree of an arbitrary node *i* is defined as the number of its neighbors, namely


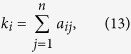


where *a*_*ij*_ is the element of matrix **A**. Degree centrality is widely applied for its simplicity and low computational cost, which works especially well in evaluating nodes’ spreading influences when the spreading rate is small.

The main idea of eigenvector centrality is that a node’s importance is not only determined by itself, but also affected by its neighbors’ importance^29^. Accordingly, eigenvector centrality of node *i*, *v*_*i*_, is defined as


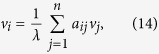


where *λ* is a constant. Obviously, Eq. (13) can be written in a compact form as


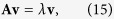


where 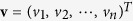. That is to say, **v** is the eigenvector of the adjacency matrix **A** and *λ* is the corresponding eigenvalue. Since the considered network is a simple undirected connected network and thus the adjacency matrix **A** is irreducible. According to Perron-Frobenius Theorem^52^, the elements in the leading eigenvector are strictly positive. Since the influences of nodes should be strictly positive, **v** must be the leading eigenvector corresponding to the largest eigenvalue of **A**, say **v** = **q**_1_.

Kitsak *et al.*^14^ argued that *k*-shell index (i.e., coreness) is a better index than degree to locate the influential nodes. The *k*-shell can be obtained by the so-called *k*-core decomposition^53^. The *k*-core decomposition process is initiated by removing all nodes with degree *k* = 1. This causes new nodes with degree *k* ≤ 1 to appear. These are also removed and the process is continued until all remaining nodes are of degree *k* > 1. The removed nodes (together with associated links) form the 1-shell, and their *k*-shell indices are all one. We next repeat this pruning process for the nodes of degree *k* = 2 to extract the 2-shell, that is, in each step the nodes with degree *k* ≤ 2 are removed. We continue with the process until we have identified all higher-layer shells and all network nodes have been removed. Then each node *i* is assigned a *k*-shell index *c*_*i*_.

### Kendall’s Tau

For each node *i*, we denote *y*_*i*_ as its spreading influence and *z*_*i*_ the target centrality measure (e.g., degree, *k*-shell index, eigenvector centrality and DS centrality), the accuracy of the target centrality in evaluating nodes’ spreading influences can be quantified by the Kendall’s Tau^36^, as


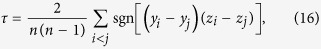


where sgn(*y*) is a piecewise function, when *y* > 0, sgn(*y*) = +1; *y* < 0, sgn(*y*) = −1; when *y* = 0, sgn(*y*) = 0. *τ* measures the correlation between two ranking lists, whose value is in the range [−1, 1] and the larger *τ* corresponds to the better performance.

## Data Description

Four real networks are studied in this paper as follows. (i) Erdös, a scientific collaboration network, where nodes are scientists and edges represent the co-authorships. The data set can be freely downloaded from the web site http://wwwp.oakland.edu/enp/thedata/. (ii) Email^54^, which is the email communication network of University Rovira i Virgili (URV) of Spain, involving faculty members, researchers, technicians, managers, administrators, and graduate students. (iii) Router^55^, the Internet at the router level, where each node represents a router and an edge represents a connection between two routers. (iv) Protein^56^, an initial version of a proteome-scale map of human binary protein-protein interaction. Basic statistical properties of the above four networks are presented in Table 1.

## Additional Information

**How to cite this article**: Liu, J.-G. *et al.* Locating influential nodes via dynamics-sensitive centrality. *Sci. Rep.* **6**, 21380; doi: 10.1038/srep21380 (2016).

## Supplementary Material

Supplementary Information

## Figures and Tables

**Table 1:** Basic statistical features of Erdös, Email, Router and Protein networks, including the number of nodes *n*, the number of the edges *e*, the average degree 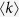 and the reciprocal of the largest eigenvalue 1/*λ*_1_.

Network	*n*	*e*	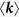	1/*λ*_1_
Erdös	454	1313	5.784	0.079
Email	1133	5451	9.622	0.048
Router	2114	6632	6.274	0.036
Protein	2783	6007	4.317	0.063
