# Supplementary material for: Locating influential nodes via dynamics-sensitive centrality
Source: Sci Rep. 2016 Feb 24;6:21380. doi: 10.1038/srep21380 (PMC4764903; doi:10.1038/srep21380)
Supplement: Supplementary Information [file srep21380-s1.pdf]

SUPPLEMENTARY INFORMATION

Locating influential nodes via dynamics-sensitive centrality

Jian-Guo Liu[1,2]*, Jian-Hong Lin[2], Qiang Guo[2], Tao Zhou[3]*

[1]Data Science and Cloud Service Centre, University of Electronic Science and Technology of China, Shanghai 200433, PR China;
[2]Research Center of Complex Systems Science, University of Shanghai for Science and Technology, Shanghai 200093, PR China;
[3]CompleX Lab, Web Sciences Center, University of Electronic Science and Technology of China, Chengdu 611731, PR China.

1 Results for Larger β

Figure S1 shows the accuracy of four centrality measures in estimating nodes' influences when the spreading rate varies from 0.11 to 0.20. Both the results for SIR model and SI model suggest that the DS centrality performs better than the other three well-known indices.

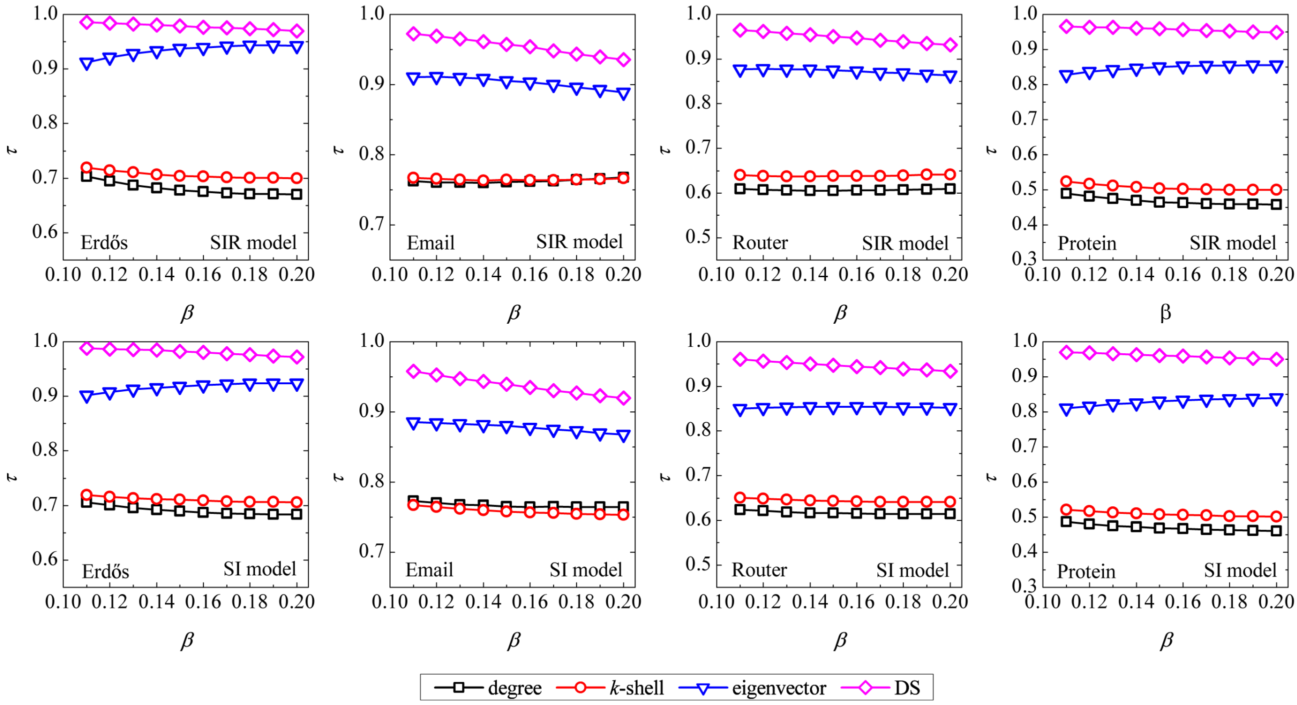

Figure S1. The accuracy of four centrality measures in evaluating nodes' spreading influences according to the SIR model ($\mu = 1$) and SI model ($\mu = 0$) in the four real networks, quantified by the Kendall's Tau. The spreading rate β varies from 0.11 to 0.20, and the time step is set as $t = 5$. Each data point is obtained by averaging over 10^4 independent runs.

2 Results for Larger t

Figure S2 shows the accuracy of four centrality measures in estimating nodes' influences when the spreading rate varies from 0.01 to 0.10, and the time step is fixed as $t = 10$. Both the results for SIR model and SI model suggest that the DS centrality performs better than the other three

well-known indices. Notice that, in comparison to the results in figures 1 and 2 in the main text, the difference between DS centrality and eigenvector centrality gets smaller.

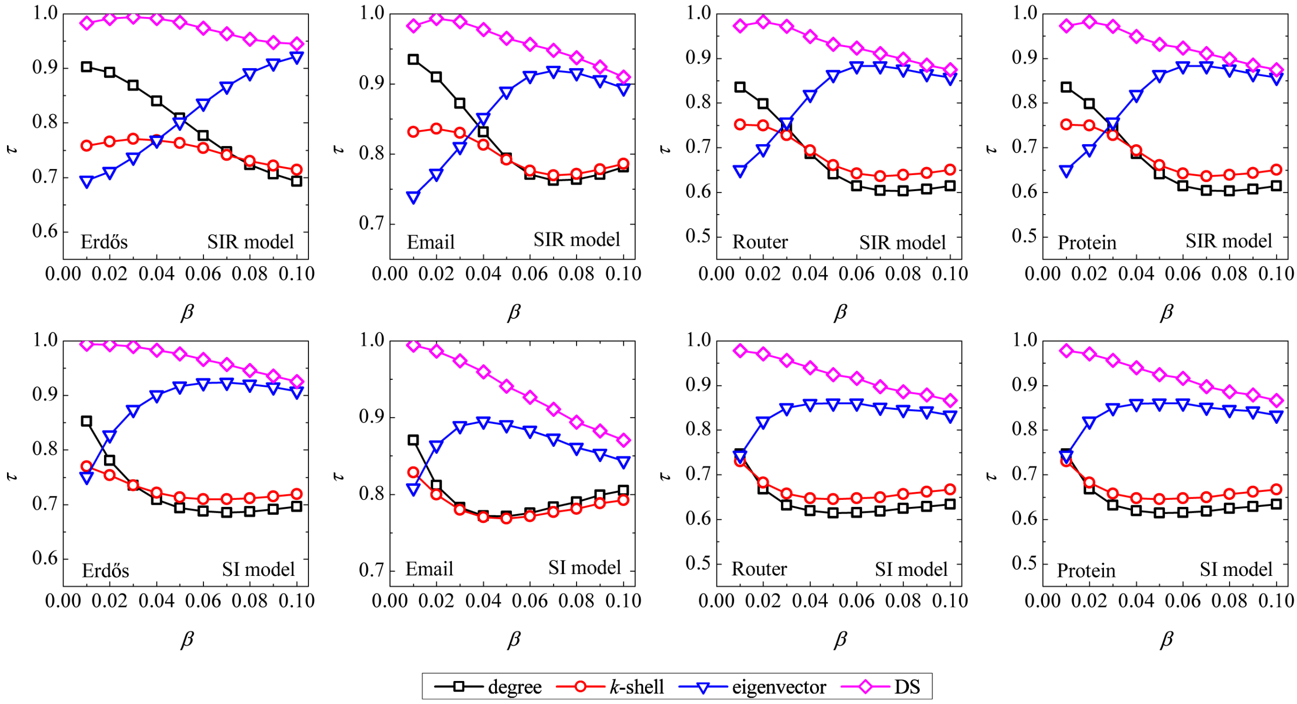

Figure S2. The accuracy of four centrality measures in evaluating nodes' spreading influences according to the SIR model ($\mu = 1$) and SI model ($\mu = 0$) in the four real networks, quantified by the Kendall's Tau. The spreading rate β varies from 0.01 to 0.10, and the time step is set as $t = 10$. Each data point is obtained by averaging over 10^4 independent runs.

3 Results for $\mu = 0.3$ and $\mu = 0.7$

Figure S3 shows the accuracy of four centrality measures in estimating nodes' influences when the spreading rate varies from 0.01 to 0.10, and the recovering rate is fixed as $\mu = 0.3$ and $\mu = 0.7$. Both the results for $\mu = 0.3$ and $\mu = 0.7$ in SIR model suggest that the DS centrality also performs better than the other three well-known indices.

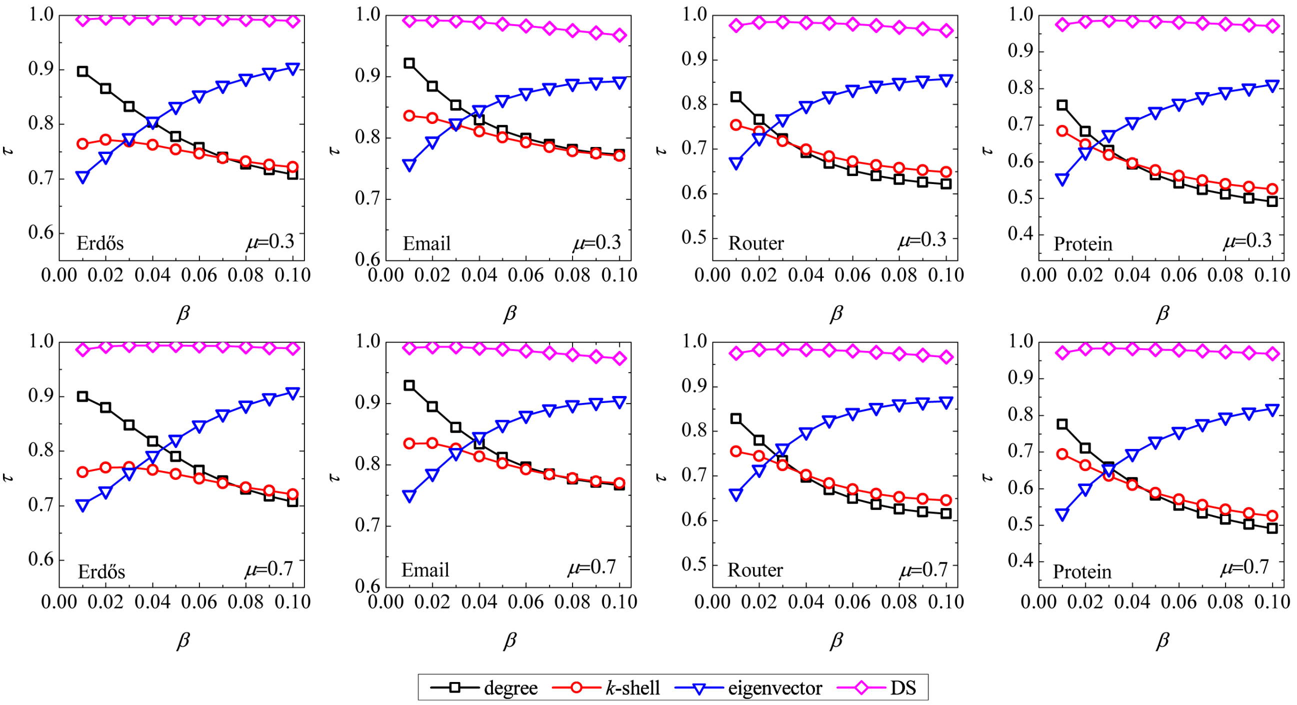

Figure S3. The accuracy of four centrality measures in evaluating nodes' spreading influences according to the SIR model for $\mu = 0.3$ and $\mu = 0.7$ in the four real networks, quantified by the Kendall's Tau. The spreading rate β varies from 0.01 to 0.10, and the time step is set as $t = 5$. Each data point is obtained by averaging over 10^4 independent runs.
